# High expression of β-catenin contributes to the crizotinib resistant phenotype in the stem-like cell population in neuroblastoma

**DOI:** 10.1038/s41598-017-17319-9

**Published:** 2017-12-04

**Authors:** Abdulraheem Alshareef, Nidhi Gupta, Hai-Feng Zhang, Chengsheng Wu, Moinul Haque, Raymond Lai

**Affiliations:** 1 Department of Laboratory Medicine and Pathology, University of Alberta, Edmonton, Alberta Canada; 2 Department of Applied Medical Sciences, Taibah University, Almedinah, P.O. Box 41477 Saudi Arabia; 3 Department of Oncology, University of Alberta, Edmonton, Alberta Canada; 4 DynaLIFE Medical Laboratories, Edmonton, Alberta Canada

## Abstract

ALK has been identified as a novel therapeutic target in neuroblastoma (NB), but resistance to ALK inhibitors (such as crizotinib) is well recognized. We recently published that the crizotinib sensitivity in NB cells strongly correlates with the crizotinib—ALK binding, and β-catenin effectively hinders this interaction and confers crizotinib resistance. Here, we asked if these observations hold true for the stem-like cells in NB cells, which were purified based on their responsiveness to a Sox2 reporter. Compared to bulk, reporter unresponsive (RU) cells, reporter responsive (RR) cells had significantly higher neurosphere formation ability, expression of *CD133/nestin* and chemo-resistance. Using the cellular thermal shift assay, we found that RR cells exhibited significantly weaker crizotinib—ALK binding and higher crizotinib resistance than RU cells. The suboptimal crizotinib—ALK binding in RR cells can be attributed to their high β-catenin expression, since siRNA knockdown of β-catenin restored the crizotinib—ALK binding and lowered the crizotinib resistance to the level of RU cells. Enforced expression of β-catenin in RU cells resulted in the opposite effects. To conclude, high expression of β-catenin in the stem-like NB cells contributes to their crizotinib resistance. Combining β-catenin inhibitors and ALK inhibitors may be useful in treating NB patients.

## Introduction

Neuroblastoma (NB) is the most common extra-cranial malignancy and the leading cause of cancer-related deaths in children^1,2^. Despite recent advances in chemotherapy and surgical care, the 5-year survival for patients with high-risk NB is less than 40%^1,2^. It is believed that NB originates from the neuro-ectodermal precursor cells derived from the neural crest; accordingly, NB tumours are typically located along the sympathetic nervous system chain^3^. The clinical course of NB patients is highly variable, and some of the most important clinicopathologic parameters used for risk stratification include patient age at diagnosis, clinical stage and tumour histology^3^. Moreover, specific genetic alterations including *MYCN* amplification, deletion of *1p36* and gain of *17q*, have been associated with a worse clinical outcome^2^. Recent studies also have highlighted the importance of intra-tumoral heterogeneity and the existence of cancer stem cells as important factors contributing to the treatment failure in NB patients^4^. Cancer stem cells are known to be highly chemo-resistant, and this phenotype is believed to be attributed to their self-renewal capability, resistance to DNA damage and other apoptosis-inducing stimuli, as well as their relatively poor accumulation of cytotoxic drugs^5^. In NB tumours, it has been shown that cancer stem cells can be identified and purified by virtue of their expression of a number of cell-surface markers, with CD133 and nestin used most frequently^6,7^.

*Anaplastic lymphoma kinase (ALK)*, which encodes a tyrosine kinase, was initially discovered and characterized in anaplastic large cell lymphoma carrying the characteristic reciprocal chromosomal translocation, *t(2;5)* ^8^. With this chromosomal translocation, the catalytic domain of the ALK protein fuses with the amino terminus of nucleophosmin (NPM), and the resulted NPM-ALK fusion protein has been found to contribute to the tumorigenicity of anaplastic large cell lymphoma by deregulating a host of cell signaling and biochemical pathways^9^. In normal cells, the expression of ALK is restricted to embryonic neuronal cells where activation of this tyrosine kinase promotes cell proliferation, survival and differentiation^10^. In NB cells, where ALK expression is frequently found, many studies have provided evidence that ALK also promotes cell growth^3^. In support of its importance, a high expression of ALK detectable by immunohistochemistry has been reported to significantly correlate with a poor clinical outcome in NB patients^11–14^. Nonetheless, the pathogenetic role of ALK in these tumors remains to be incompletely understood. To date, the oncogenic role of ALK in NB cells is best defined by studying the biological effects of specific *ALK* mutations localized in its tyrosine kinase domain^15–18^. In this regard, three mutation sites present in the tyrosine kinase domain (i.e. 1174, 1245 and 1275) were found to account for 85% of all *ALK* missense mutations in NB^19^. The oncogenic potential of ALK^F1174L^ has been the most studied, as this *ALK* mutant was found to exert potent oncogenic effects in both *in-vitro* and *in-vivo* models^20^. In keeping with the importance of this *ALK* mutation, patients with tumors carrying *ALK* mutation at residue 1174 were found to have a poor clinical outcome^19^. In view of these observations, crizotinib, the first ALK inhibitor approved for clinical use, was tested to treat NB patients with recurrent or refractory diseases in a phase 1 clinical trial^21^. Unfortunately, the overall clinical response to crizotinib was suboptimal, with only 2 of 34 (6%) patients showing complete remission^21^. In fact, this clinical observation correlates with the results of several *in-vitro* studies, which found that NB cell lines display a wide range of crizotinib sensitivity, with the IC_50_ (i.e. inhibitory concentration at 50%) ranging from 10 to > 3000 nM^19,22,23^. With respect to ALK^F1174L^, it has been shown that this specific mutation can increase the affinity for ATP at the expense of crizotinib^19^, but ALK^F1174L^-carrying cell lines displayed drastically different IC_50_ to crizotinib (i.e. IC_50_, 400 to 2000 nM)^24^. Overall, the mechanism underlying the crizotinib resistance in NB cells is incompletely understood.

We have recently published evidence that the physical interaction between ALK and crizotinib is an important determinant of crizotinib sensitivity in NB cells, and this interaction may be affected by the mutational status of *ALK*. Furthermore, β-catenin, a binding partner of ALK in NB cells, can provide significant hindrance to the crizotinib—ALK binding, especially when it is highly expressed^22^. In this study, we asked if these observations hold true for cancer stem-like cell population in NB. Stem-like NB cells were purified based on their responsiveness to a Sox2 reporter, a strategy previously used for several different cancer models^25–30^.

## Results

### Identification of two cell subsets based on their differential response to a Sox2 reporter

**Figure 1:**
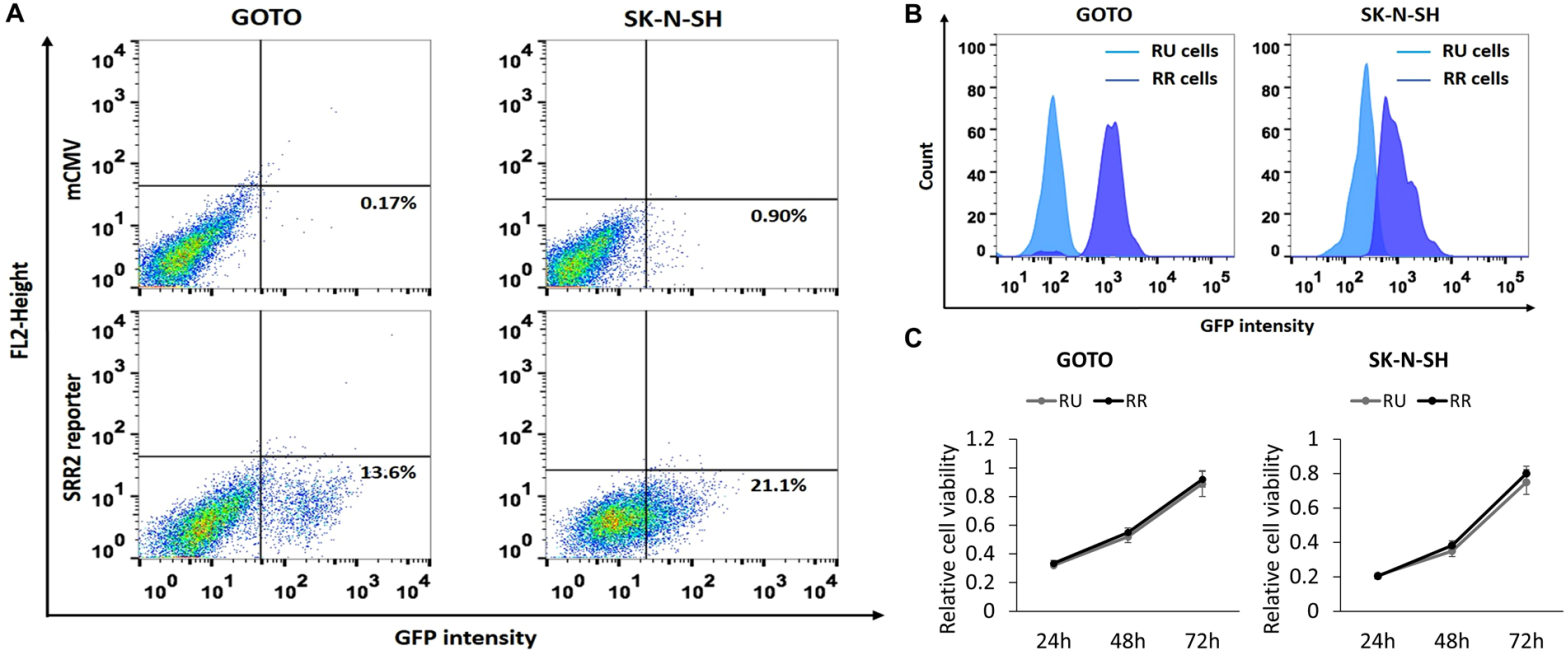
Identification of two cell subsets in NB cell lines. (**A**) Flow cytometry analysis was performed to detect GFP expression in NB cells stably infected with the lentiviral SRR2 reporter. In both cell lines, a small subset of reporter responsive (RR) cells was found, whereas the majority of the cells were unresponsive to the reporter (i.e. reporter unresponsive, RU cells). NB cells stably infected with the mCMV (negative control) lentivirus were used as the negative control. (**B**) GFP expression in purified RU and RR cells purified from cells stably infected with the lentiviral SRR2 reporter were substantially different. (**C**) Cell growth of RU and RR cells were assessed by using the MTS assay. All data are presented as mean ± SD, *p < 0.05, Student’s *t* test. Abbreviations: NB, neuroblastoma; SRR2, Sox2 regulatory region 2; mCMV: Murine Cytomegalovirus; GFP: Green Fluorescence Protein.

Using a commercially available lentiviral Sox2 reporter, we have previously identified a novel intra-tumoral dichotomy in various types of human cancer^25–28^. In this study, we asked if the same intra-tumoral dichotomy also can be identified in NB cells. Based on their expression of GFP, Reporter Responsive (RR) cells were detectable by flow cytometry in 2 of 2 NB cell lines (GOTO and SK-N-SH) examined. As shown in Fig. 1A, we found that RR cells accounted for 13.6% and 21.1% in GOTO and SK-N-SH, respectively. Cells infected with the empty vector (labelled as *mCMV*) were used as the negative controls.

To further study the biological significance of this intra-tumoral dichotomy, we purified RR cells and Reporter Unresponsive (RU) cells derived from both cell lines using a flow cytometric cell sorter, and these subsets were cultured separately. The differential GFP expression levels between purified RU and RR cells are illustrated in Fig. 1B. As shown in Fig. 1C, purified RU and RR cells derived from these two cell lines had no significant difference in the growth rate. We also confirmed that the gene copy number of the Sox2 reporter integrated into these 2 cell subsets was not significantly different (data not shown), and thus, the difference in their reporter response was genuine. Lastly, since RR cells were found to lose GFP expression gradually (i.e. approximately 25% in 4 weeks), we purified RR cells immediately before each of the following experiments. In contrast, we did not find evidence that purified RU cells can convert into RR cells. As shown in Supplementary Figure 1, there was no emergence of GFP-positive cells in purified RU cells derived from GOTO and SK-N-SH cultured for 10 weeks.

### RR cells are more stem-like and chemo-resistant than RU cells

**Figure 2:**
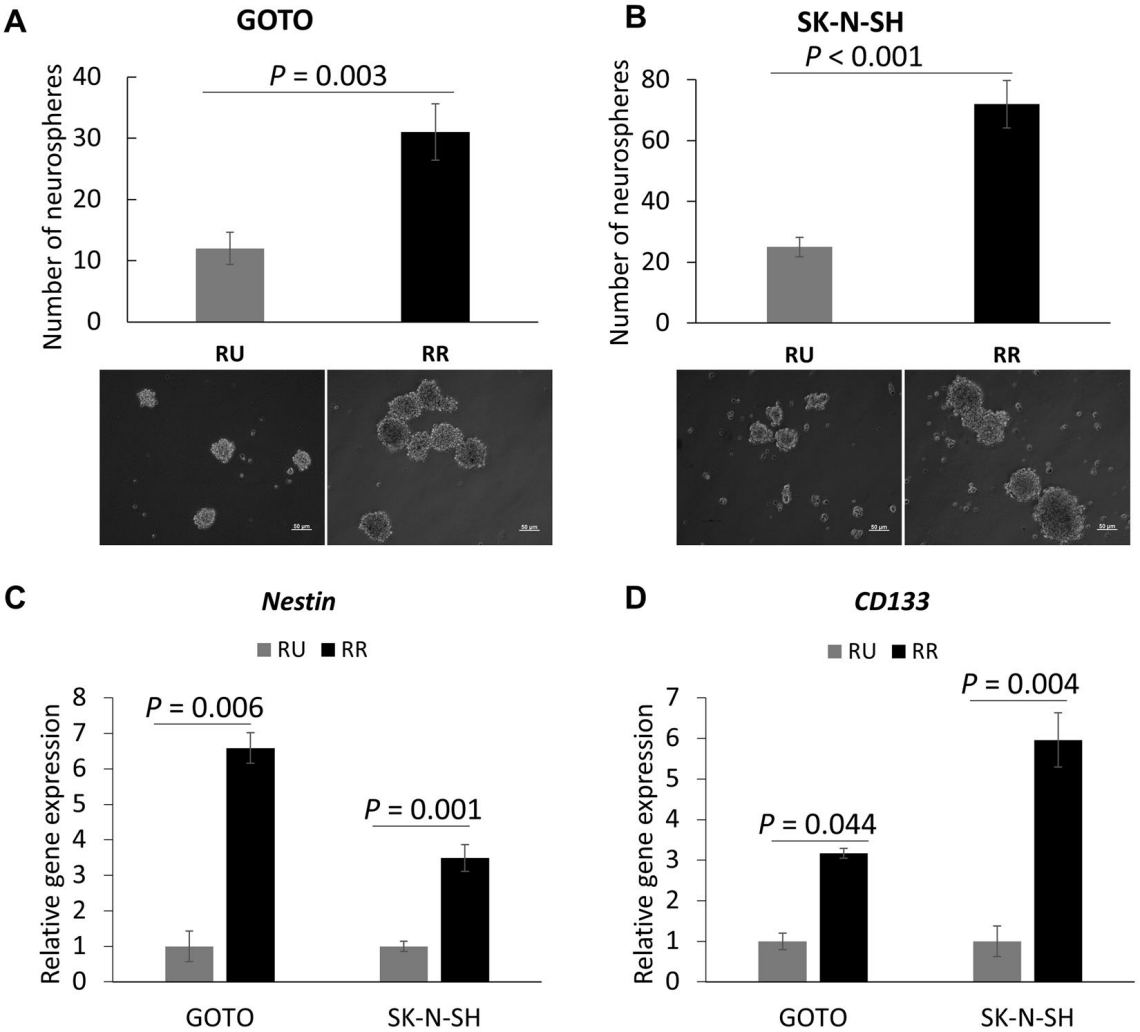
RR cells carry more stem-like features compared to RU cells. (**A**,**B**) Neurosphere formation assay was performed to compare the stem-like properties between RU and RR cells. Images were taken at x100 magnification. (**C**,**D**) *Nestin* and *CD133* mRNA expressions in RU and RR cells were examined using quantitative RT-PCR. All data are presented as mean ± SD. Student’s *t* test was performed.

To assess the biological significance of the identified RU/RR dichotomy, we performed a number of functional assays to compare RU and RR cells. First, we compared these two cell subsets with respect to their cancer stem-like features using the neurosphere formation assay. As shown in Fig. 2A,B, we found that RR cells demonstrated a significantly higher capacity to form neurospheres than RU cells (~3 folds, *p* < 0.005) in both NB cell lines. Correlating with this finding, RR cells derived from both cell lines contained a significantly higher proportion of cells expressing *nestin* and *CD133*, as measured by using quantitative RT-PCR (Fig. 2C,D). Of note, both nestin and CD133 have been used to detect and purify cancer stem cells in NB^31^.

We then compared the sensitivity of RU and RR cells to doxorubicin and cisplatin, two chemotherapeutic agents used to treat NB patients^32^. We found that RR cells derived from both cell lines showed a significantly higher IC_50_ (inhibitory concentration at 50%) to doxorubicin than RU cells (GOTO, 2 μM versus 0.71 μM, *p* < 0.001; SK-N-SH, 1.2 μM versus 0.51 μM, *p* < 0.001) (Supplementary Figure 2A and 2B). Similarly, RR cells showed a significantly higher IC_50_ to cisplatin compared to RU cells (GOTO, 11.6 μM versus 5.8 μM, *p* < 0.001; SK-N-SH, 8.6 μM versus 4.1 μM, *p* < 0.001) (Supplementary Figure 2C and 2D).

Next, we compared RU and RR cells with respect to their resistance to oxidative stress, which is known to be higher in normal stem cells and cancer stem cells^33^. By subjecting RU and RR cells to increasing concentrations of H_2_O_2_, a potent inducer of oxidative stress^33^, we found that RR cells were significantly more resistant than RU cells (IC_50_ for GOTO, 319 μM versus 192 μM, *p* < 0.001; IC_50_ for SK-N-SH, 495 μM versus 185 μM, *p* < 0.001) (Supplementary Figure 2E and 2F). Taken together, RR but not RU cells are enriched with cancer stem-like cells in NB.

### RR cells are resistant to crizotinib, an ALK inhibitor

**Figure 3:**
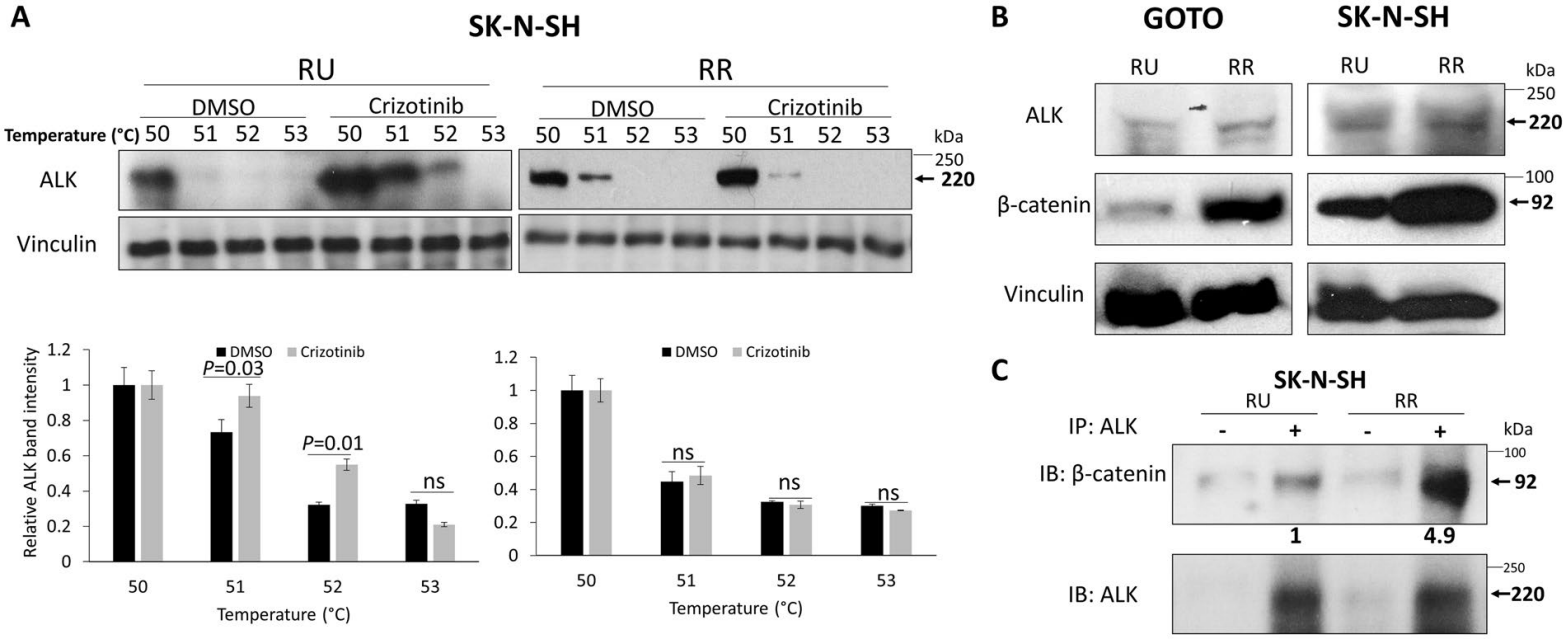
RR cells demonstrate no crizotinib—ALK binding and higher expression level of β-catenin compared to RU cells. (**A**) CETSA was performed to compare crizotinib—ALK binding ability between RU and RR cells. RU and RR cells derived from SK-N-SH were treated with DMSO or 500 nM crizotinib for 6 hours. Representative ALK western blots are shown on the upper panel. Vinculin level was blotted as a loading control. The densitometry quantification data from 3 independent experiments are shown on the lower panel. All data are presented as mean ± SD. Student’s *t* test was performed. (**B**) The expression of ALK and β-catenin protein in RU and RR cells were measured by western blots. Vinculin level was blotted as a loading control. (**C**) ALK pull-down was performed using co-immunoprecipitation assay and showed substantial ALK-β-catenin binding in RR cells compared to RU cells.

ALK has been recognized as one of the most promising druggable targets identified in NB^34^. Thus, we assessed if RR and RU cells have differential sensitivity to crizotinib, the first ALK inhibitor approved for clinical use^34^. RR cells were found to be significantly more resistant to crizotinib than RU cells, with the IC_50_ for these two cell subsets being significantly different (GOTO, 1989 nM versus 822 nM, *p* < 0.001; SK-N-SH, 724 nM versus 452 nM, *p* < 0.001) (Supplementary Figure 2G and 2H). Since we have recently published that the crizotinib sensitivity in NB cells is linked to the crizotinib—ALK binding^22^, we performed the cellular thermal shift assay (CETSA), a method shown to be useful in quantifying drug-target interactions^35–37^. As detailed previously^22^, the crizotinib—ALK binding was assessed ‘strong’ if crizotinib-treated cells contain a significantly higher level of ALK protein than DMSO-treated cells (i.e. negative control) at 52 °C. As shown in Fig. 3A, using western blots, we identified strong crizotinib—ALK binding in RU cells derived from SK-N-SH at 52 °C, at which no substantial crizotinib—ALK binding was identified in RR cells. Similar results were observed in RU and RR cells derived from GOTO (Supplementary Figure 3). These findings correlate well with our observation that RR cells were more resistant to crizotinib than RU cells.

### β-catenin contributes to the differential crizotinib sensitivity between RU and RR cells

We have previously shown that the β-catenin—ALK interaction contributes to the weak crizotinib—ALK binding and increases the crizotinib resistance in NB cells^22^. Therefore, we asked if the β-catenin—ALK interaction also plays a role in generating the differential crizotinib sensitivity between RU and RR cells. As shown in Fig. 3B, RR cells showed a substantially higher β-catenin protein level compared to RU cells. In contrast, the ALK protein level was only slightly different between RU and RR cells in both NB cell lines. By immunoprecipitation, we found that the β-catenin—ALK interaction was more abundant in RR cells (~5-fold) relative to RU cells (Fig. 3C).

**Figure 4:**
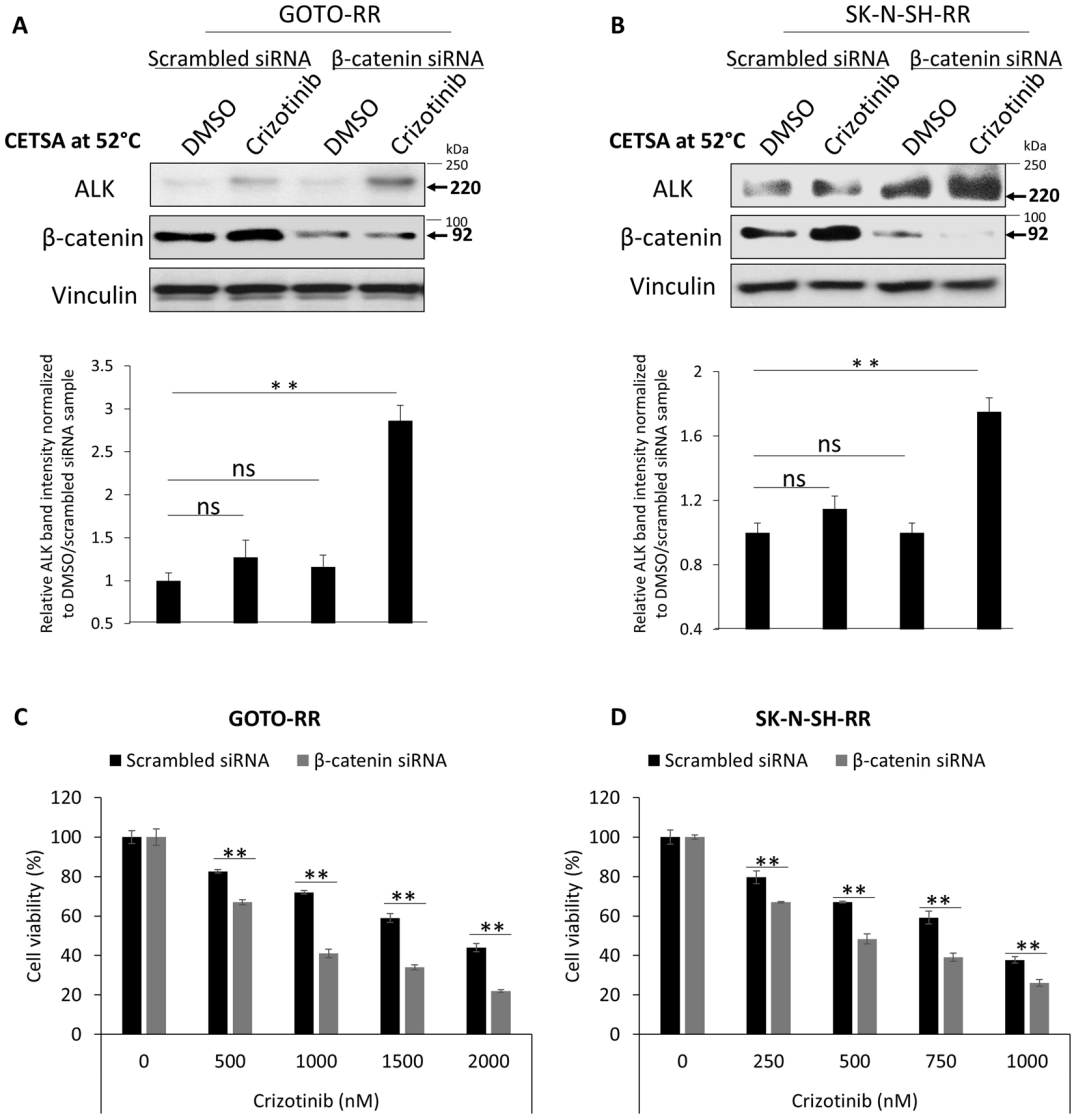
β-catenin siRNA knockdown restores the binding between crizotinib and ALK and significantly sensitizes RR cells to crizotinib treatment. (**A**,**B**) β-catenin siRNA knockdown significantly restores crizotinib—ALK binding upon crizotinib treatment in comparison to scrambled siRNA transfection in RR cells derived from GOTO and SK-N-SH. Representative western blots are shown on the upper panel and the densitometry quantification data from 3 independent experiments are shown on the lower panel. (**C**,**D**) RR cells significantly sensitized to crizotinib treatment upon β-catenin siRNA knockdown. Data are presented as mean ± SD. **P < 0.01, Student’s *t* test.

To directly establish the link between the β-catenin—ALK interaction and the resistance to crizotinib in NB cells, we subjected RR cells derived from GOTO and SK-N-SH to β-catenin siRNA knockdown, followed by the CETSA. As shown in Fig. 4A,B, restoration of the crizotinib—ALK binding was observed upon β-catenin siRNA knockdown. The siRNA treatment also conferred a significant sensitization to crizotinib, with the IC_50_ decreased from 1951 nM (i.e. treated with scrambled siRNA) to 988 nM in GOTO cells (Fig. 4C). The IC_50_ of RR cells transfected with β-catenin siRNA approached that of native RU cells (i.e. 822 nM). As for SK-N-SH cells, the IC_50_ of RR cells decreased from 804 nM (negative control) to 472 nM, and this level was comparable to that of native RU cells (i.e. 452 nM) (Fig. 4D). Of note, β-catenin siRNA knockdown alone (for 72 hours) did not significantly affect the cell growth of both cell lines (data not shown).

**Figure 5:**
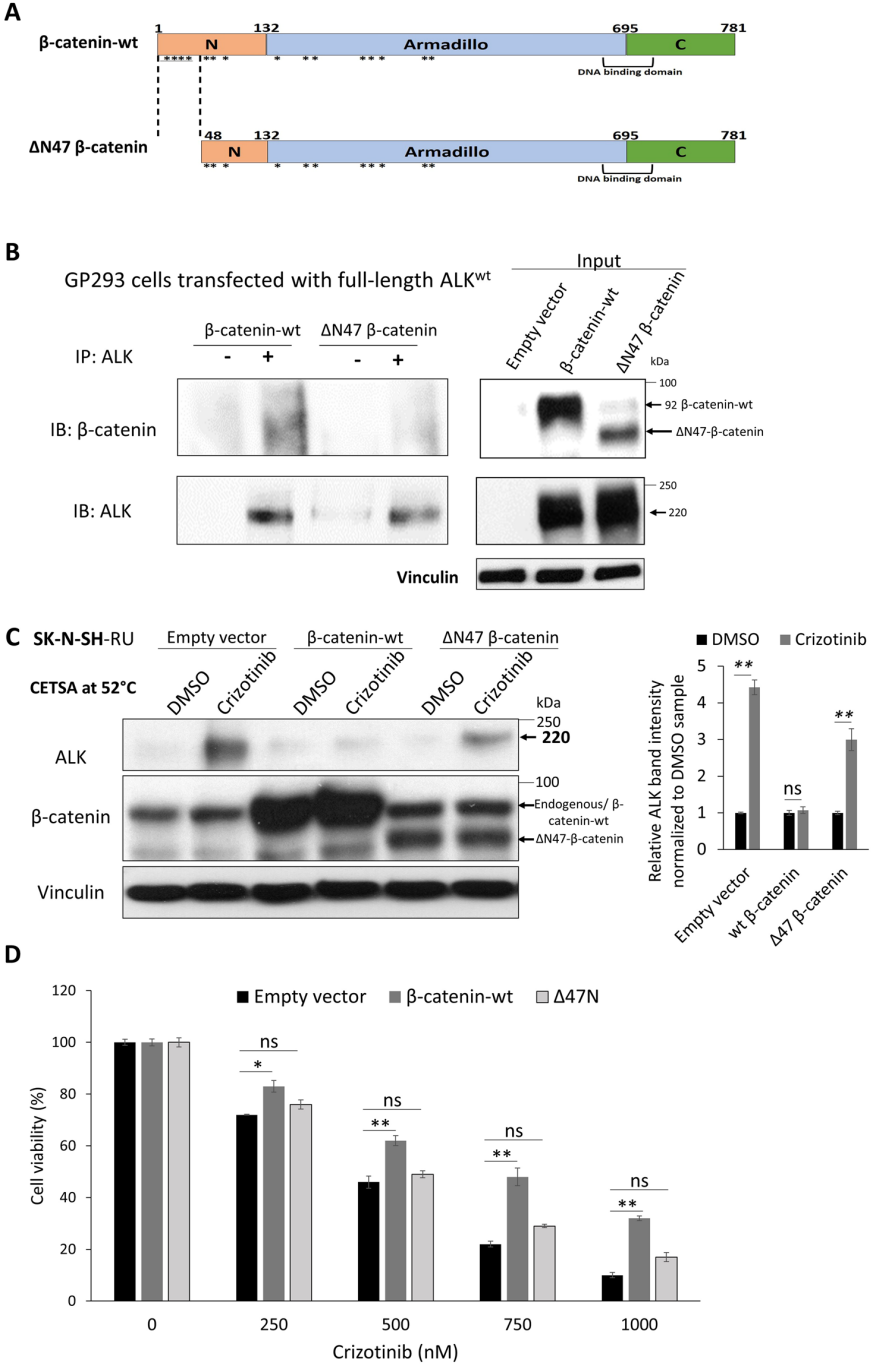
Enforced β-catenin-wt interacts with ALK and abrogates crizotinib—ALK binding in SK-N-SH-RU cells. (**A**) Diagram of the two *β-catenin* plasmids used in this study. Locations of computationally predicted β-catenin residues interacting with ALK (16 residues, *). (**B**) ALK pull-down was performed using co-immunoprecipitation assay in GP293 cells that were transfected with full-length *ALK* and with either *β-catenin-*wt or ΔN47*-β-catenin*. Full-length *ALK* co-immunoprecipitated with wild-type β-catenin but not ΔN47 β-catenin. Another exposure for the co-immunoprecipitation blots are presented in Supplementary Figure 5. (**C**) CETSA was performed to compare crizotinib—ALK binding ability in RU that were transfected with either empty vector, β-catenin-wt or ΔN47-β-catenin. Cells were treated with DMSO or 500 nM crizotinib for 6 hours. Representative ALK western blots are shown on the left panel. CETSA assay was performed at 52 °C. Vinculin level was blotted as a loading control. (**D**) Enforced expression of β-catenin-wt but not ΔN47 significantly affect crizotinib sensitivity in RU cells in comparison to empty vector transfected cells. All data are presented as mean ± SD, *P < 0.05, **P < 0.01, Student’s *t* test.

Next, we used a *β-catenin* mutant that carries a deletion of the first 47 amino acids in the N-terminal region (ΔN47)^38^. The particular segment of β-catenin was chosen based on the results of our prior computational analysis of the β-catenin—ALK interaction^22^. Specifically, we predicted a total of 16 residues of β-catenin are involved in the β-catenin—ALK interaction, as illustrated in Fig. 5A. By deleting the first 47 amino acid residues in the N-terminal of β-catenin, we were able to remove 4 (25%) of these 16 residues. Furthermore, the N-terminal was chosen because it is farthest away from the β-catenin DNA binding domain (Fig. 5A) such that the transcription activity of β-catenin is likely preserved. As shown in Fig. 5B, a substantial amount of wild-type *β-catenin* (*β-catenin-wt*) and ΔN47 *β-catenin* were expressed in GP293 cells (i.e. input). Using immunoprecipitation, we were able to pull down a relatively equal amount of full-length ALK in cells expressing wt or ΔN47 *β-catenin*. Under this condition, we found that full-length ALK co-immunoprecipitated with a relatively high level of *β-catenin-wt* but no appreciable level of ΔN47 *β-catenin*. Correlating with these findings, while we found that the crizotinib—ALK binding in RU cells was largely abrogated upon the enforced expression of *β-catenin-wt*, transfection of ΔN47 into RU cells did not substantially affect the crizotinib—ALK binding (Fig. 5C). Of note, transfection of ΔN89 (a construct that carry additional deletions of the β-catenin N-terminus) into RU cells also did not substantially affect the crizotinib—ALK binding (Supplementary Figure 4). Accordingly, we found that the IC_50_ of cells transfected with *β-catenin-wt* increased and approached that of native RR cells (i.e. 724 nM); in comparison, the IC_50_ of cells transfected with ΔN47did not change significantly (Fig. 5D). Taken together, we concluded that the physical interaction between β-catenin and ALK is an important factor in β-catenin—mediated resistance to crizotinib in NB cells.

### Suboptimal crizotinib—ALK binding in RR cells is not due to lack of crizotinib bioavailability

**Figure 6:**
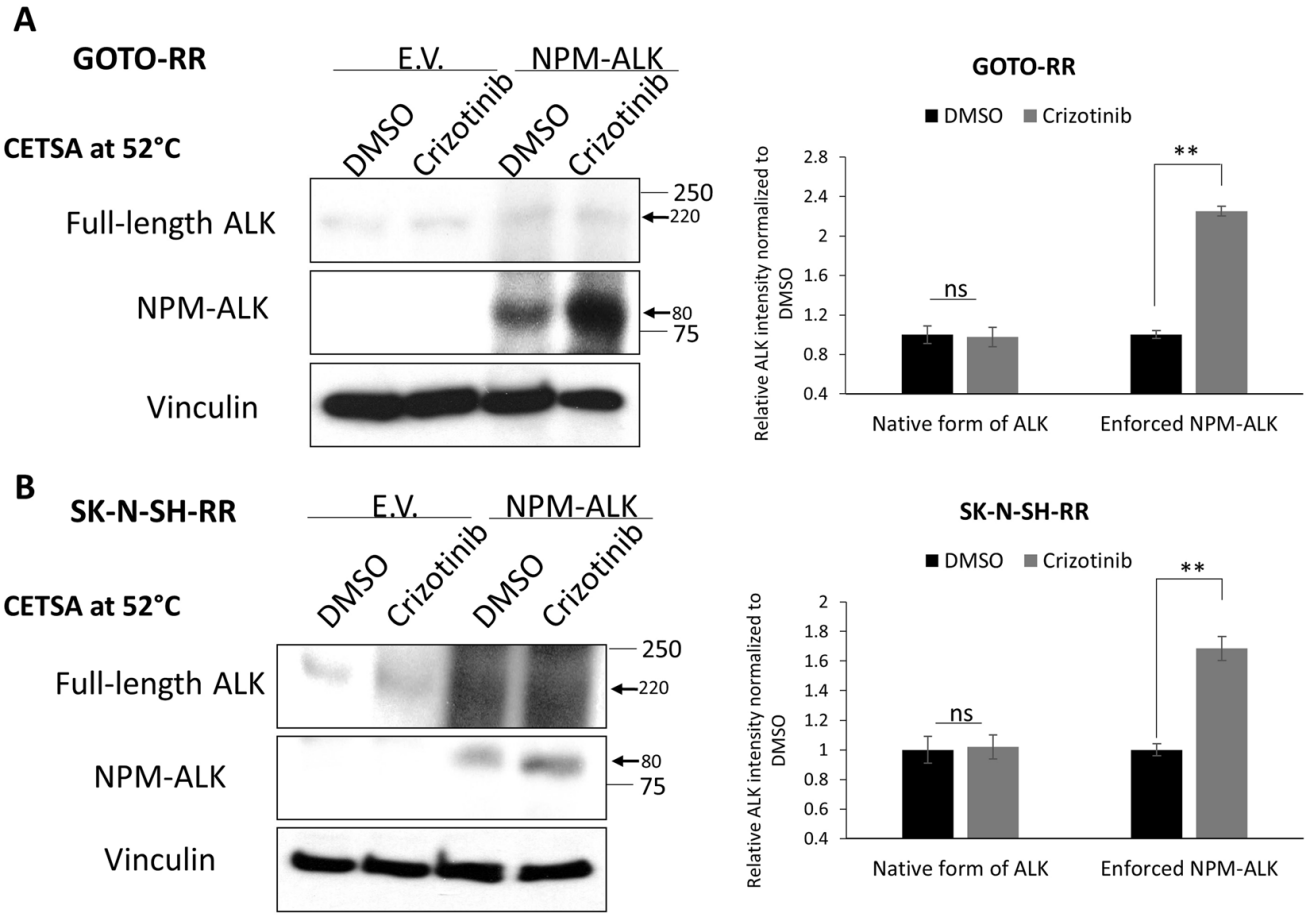
Crizotinib resistance in RR cell is not due to lack of intracellular availability of crizotinib. (**A**,**B**) Enforced expression of NPM-ALK into RR cells derived from GOTO or SK-N-SH showed crizotinib—NPM-ALK binding while no binding to the native ALK. CETSA assay was performed at 52 °C. Representative western blots are shown on the left side and the densitometry quantification data from 3 independent experiments are shown on the right side. Data are presented as mean ± SD. **P < 0.01, Student’s *t* test.

One possible explanation for the suboptimal crizotinib—ALK binding in RR cells is related to the relatively poor drug accumulation in this cell subset. To assess this possible explanation, we transiently transfected *NPM-ALK* into RR cells. By CETSA, we found substantial crizotinib—NPM-ALK binding (Fig. 6A,B). In the same experiments, there was no substantial binding between crizotinib and the native forms of ALK. Thus, the differential crizotinib—ALK binding and crizotinib sensitivity between RU and RR cells is not due to a difference in the bioavailability of crizotinib inside the cells.

## Discussion

Using a commercially available Sox2 reporter, we identified the novel RU/RR dichotomy in NB cells, with the small subset of RR cells being significantly more stem-like than RU cells. Our finding of the small subset of stem-like cells in NB cell lines is in alignment with the experimental findings from several groups, who also had successfully identified cancer stem cells in NB^5,39–47^. CD133, a transmembrane glycoprotein, is probably the marker used most frequently in identifying cancer stem cells in NB^5^. The link between CD133 and cancer stemness in NB is relatively well supported, and some of the evidence came from the observation that CD133-positive NB cells are significantly more tumorigenic in mouse xenograft models than their CD133-negative counterparts^40,43,48^. Other markers of cancer stem cells in NB also have been reported, and they include CD114, Frizzled receptor 6, ALDH1A2, as well as the ability to exclude Hoechst 33342^39–47^. In contrast with these previous studies, our method to detect stem-like cells was based on their differential responsiveness to a Sox2 reporter. To be discussed below, others and we have found this strategy to be useful and effective in identifying and purifying stem-like cells in other cancer models. In support of the validity of this experimental approach, we found that RR cells exhibited significantly higher neurosphere formation ability and chemo-resistance than RU cells. Furthermore, compared to RU cells, RR cells expressed a significantly higher level of CD133.

The identification of the stem-like cell population based on their differential responsiveness to the Sox2 reporter has been demonstrated in a number of cancer models. Using the same Sox2 reporter described in the current study, others and we have published the novel intra-tumoral RU/RR dichotomy detectable in oestrogen receptor-positive breast cancer, triple-negative breast cancer (TNBC), oesophageal squamous cell carcinoma and ALK-positive anaplastic large cell lymphoma^25–30^. In all of these study models, RR cells, which typically represent a relatively small cell subset, were found to be consistently more tumorigenic and stem-like than the RU counterparts. Thus, it is tempting to speculate that the significance of the RU/RR dichotomy and the concept that RR phenotype being a cancer stem cells marker can be generalized to other types of human cancer. While we did not include NB patient samples in the current study, we have documented the existence of the RU/RR dichotomy in tumour samples derived from ER+ breast cancer as well as TNBC^26,27^. Thus, the RU/RR dichotomy is not cell line-specific phenomenon. Overall, we believe that this is a useful experimental model to study the biology of cancer stemness, especially we have demonstrated that the RU and RR phenotype can be converted into each other by using specific experimental manipulation^49,50^.

While the stem-like cells in NB have been shown to be more resistant to conventional chemotherapeutic agents^31^, whether they are also more resistant to targeted therapy is largely unknown. Since ALK has been recently postulated to be a useful therapeutic target for NB patients, we compared the sensitivity of RU and RR cells to crizotinib, the first clinically used ALK inhibitor. We found that RR cells were significantly more resistant to crizotinib than RU cells, suggesting that cancer stem-like cells may have been a contributing factor to treatment failure and disease relapses to targeted therapy in NB patients. To this point, we have identified a small number of published studies that had pointed to the similar conclusion. In one study, it was found that the stem-like cells in chronic myeloid leukaemia cell lines are relatively resistant to imatinib, a tyrosine kinase inhibitor targeting the oncogenic fusion protein BCR–ABL^51^. In another study, the stem-like cells identified in lung cancer cell lines were found to be relatively resistant to gefitinib, a tyrosine kinase inhibitor targeting the epidermal growth factor receptor^52^. In chronic myeloid leukaemia (CML) led by the BCR-ABL fusion, β-catenin has a well-documented role in mediating resistance to tyrosine kinase inhibitors^53^. For example, the multi-drug exporter channel protein ABCB1 was identified to be a downstream target of β-catenin in CML^54^. Increased β-catenin expression/activity has been found to be a consistent feature of CML stem cells. Moreover, the activity of β-catenin was found to be essential for cancer stem cell renewal as its deletion significantly abrogated CML expansion *in vivo* ^55^.

In chronic myeloid leukaemia, several mechanisms responsible for their resistance to imatinib have been postulated. First, stem-like leukemic cells were found to express a relatively high level of BCR-ABL, thereby leading to an increase in IC_50_^56,57^. Second, the intracellular accumulation of imatinib in stem-like cells was found to be relatively inefficient, due to the low expression of OCT1 (organic cation transporter-1)^58^ as well as the high expression of ABCB1 (ATP Binding Cassette Subfamily B Member-1)^59^. Third, stem-like leukemic cells were found to have the preferential activation of pro-survival/anti-apoptotic signalling pathways such as those of MAPK, notch and hedgehog^60^. Regarding how RR cells in NB are more resistant to crizotinib, we believe that multiple mechanisms may be in place. Nonetheless, we did not find a substantial difference in the expression of ALK between RU and RR cells. We also found no evidence to support the notion that the intracellular accumulation of crizotinib is suboptimal in RR cells, since transfected *NPM-ALK* expressed in RR cells binds well to crizotinib, assessed using CETSA. Lastly, other than the differential expression of β-catenin, we did not observe substantial differences in the expression or activation status of key signalling pathways such as those of STAT3 and AKT (data not shown). Thus, the mechanisms used to increase the resistance to targeted therapeutic in stem-like cells may be cell type-specific.

To our knowledge, relatively few studies have shown that stem-like cells derived from various cancers, including NB, express a relatively high level of β-catenin^40,61–63^. While inhibition of β-catenin was found to decrease cancer stemness and tumorigenicity in some of these studies, whether β-catenin plays a direct role in this context has not been fully examined. Our findings have strongly suggested that β-catenin is a key contributory factor to the high crizotinib resistance in the NB stem-like cells. Specifically, transfection of *β-catenin-wt* in RU cells conferred resistance to crizotinib, whereas siRNA knockdown of β-catenin in RR cells led to a significant decrease in IC_50_ to the level of RU cells. Regarding how β-catenin mediates these biological effects, results from one of our recently publications have suggested that β-catenin likely hinders the binding of crizotinib to ALK^22^. This conclusion is supported by two lines of evidence. First, computational analysis has revealed that the ALK binding site recognized by β-catenin substantially overlaps with that of crizotinib^22^. Accordingly, siRNA knockdown of β-catenin in RR cells significantly increased the crizotinib—ALK binding (detectable by CETSA) and decreased the IC_50_. Second, as shown in this current study, a relatively short deletion in β-catenin (i.e. ΔN47, deletion of the first 47 amino acids out of the total 781 amino acid peptide) abrogated the β-catenin-mediated crizotinib resistance in RU cells. Results from the experiments using ΔN47 also argue against the possibility that β-catenin increased the crizotinib resistance simply due to its transcriptional activity, as its DNA binding site is remote from the deletions in ΔN47^38^.

Why RU and RR cells express different expression levels of β-catenin is unknown, but some of our observations may have provided some clues. First, we did not observe a significant difference in the mRNA level of *β-catenin* between RU and RR cells (unpublished data). This finding echoes that of another group, who did not find a substantial difference in the mRNA level of *β-catenin* between CD133-positive NB cells and CD133-negative NB cells, despite the fact that the β-catenin protein was higher in the CD133-positive subset^64^. Taken together, we believe that the differential β-catenin expression between RU and RR cells is likely generated post-translationally. Second, our recent study of ALK-positive anaplastic large cell lymphoma cells has shown that RR cells express a higher level of Wnt ligand (i.e. Wnt2B), which contribute to the higher activation status of the Wnt canonical pathway and the higher expression level of β-catenin^49^. Thus, future studies may include evaluation of the activation status of the Wnt canonical pathway in RU and RR cells.

To conclude, our study has identified a subset of stem-like cells in NB based on their differential response to a Sox2 reporter. Our studies have provided one of the few examples in which cancer stem-like cells are known to be highly resistant to targeted therapy. Knowing the role of β-catenin in crizotinib resistance in NB, we propose that combining inhibitors for ALK and β-catenin may be an effective approach to treat NB patients.

## Materials and Methods

### Cell lines and stable cell clone generation

GOTO and SK-N-SH human NB cell lines were kind gifts of Dr. Roseline Godbout (Department of Oncology, University of Alberta). Of note, GOTO cells are known to carry amplified *MYCN* and wild-type *ALK* while SK-N-SH cells are known to carry non-amplified *MYCN* and mutated *ALK* ^*F1174L*^ ^65^. Cells were cultured in RPMI-1640 medium supplemented with 10% foetal bovine serum. The RU and RR cells derived from the GOTO and SK-N-SH cell lines were generated as previously described^27,28^. Briefly, these cells were infected with lentivirus carrying the pGreenFire1-mCMV-EF1-Puro vector or pGreenFire1-mCMV-Sox2SRR2-EF1-Puro vector (SBI System Biosciences, CA, USA). The pGreenFire1-Sox2SRR2-mCMV-EF1-Puro vector contained three tandem repeat of Sox2 regulatory region 2 (SRR2), which is 5′-AAAGAATTTCCCGGGCTCGGGCAGCCATTGTGATGCATATAGGATTATTC-ACGTGGTAATG-3′. The underlined sequence is the Sox2 consensus sequence. Stable cell clones were selected for two weeks in media containing 2 μg/ml Puromycin. To isolate the RR and RU cell clones, ~10% cells showing the highest/lowest GFP were isolated respectively using fluorescence activated cell sorting (FACS) as described previously^27,28^. All the stable cell clones derived from these cells were cultured in the same type of medium that was used for the parental cell line. Parental cells and stable cell clones have been authenticated using short tandem repeat DNA profiling (from TCAG Genetic Analysis Facility, Toronto, CA).

### Neurosphere formation assay

After trypsinization, single-cell suspensions were obtained by filtering the cells using a 40 µm Cell Strainer (BD Biosciences). Then, cells were counted and 500 cells were grown in NeuroCult complete media consisting of NeuroCult Neural Stem Cell (NSC) Basal medium, 1/10 NeuroCult NSC Proliferation supplements, 20 ng/ml EGF, 10 ng/ml bFGF, and 2 μg/ml Heparin. NeuroCult media, supplements, and growth factors were all purchased from Stem Cell Technologies (Vancouver, BC, Canada). Cells were cultured for two weeks before the neurosphere containing more than twenty cells were counted.

### RNA isolation and quantitative real-time PCR (qRT-PCR)

Total RNA was extracted from RU and RR cell subsets with the RNeasy Mini Kit (Qiagen, Valencia, CA). Trace DNA was removed by treatment with TURBO DNA-free Kit (Ambion, Life Technologies, Carlsbad, California, USA). Reverse Transcription (RT) reactions were performed with 1 μg of total RNA using Superscript First-Strand Synthesis System Kits (Invitrogen, Carlsbad, CA). The sequences of the primers used in this study include: Primer set for *CD133* (*PROM1*): (F-AGTCGGAAACTGGCAGATAGC) & (R-GGTAGTGTTGTACTGGGCCAAT), primer set for *nestin* (*NES*): (F-GTGGCTCCAAGACTTCC) & (R-GCACAGGTGTCTCAAGG) and primer set for GAPDH: (F- GGTCTCCTCTGACTTCAACAGCG) & (R- ACCACCCTGTTGCTGTAGCCAA). GAPDH was used as an internal control.

### Chemo-resistance assay

Cells were plated in 96-well plates, 2000 cells/well. 24 h after plating, cells were treated with different doses of Cisplatin or Doxorubicin under normal culture conditions. Cell viability was measured using MTS assay (Promega) 3 days after drug treatment.

### Cellular thermal shift assay (CETSA)

The ability of crizotinib to interact with, and thereby stabilize ALK in intact cells, was analysed essentially as described by Molina *et al*.^35^. Briefly, cells cultured in 100 × 20 mm tissue culture dishes at 90% confluence were treated with media containing DMSO or crizotinib (doses used as described in the text) for 6 hours. After treatment, cells were detached with trypsin, collected by centrifugation and subsequently resuspended in PBS. The cell suspension was aliquoted into four PCR tubes and heated for 3 minutes to 50, 51, 52 or 53 °C. Subsequently, cells were lysed using liquid nitrogen and two repeated cycles of freeze-thaw. Precipitated proteins were separated from the soluble fraction by centrifugation at > 17,000 g for 15 minutes. Soluble proteins, collected in the supernatant, were kept at −80 °C until Western blot analysis. Equal amount of proteins were loaded onto 6% SDS–PAGE gels, transferred to nitrocellulose membranes and analysed using the ALK-antibody from Cell Signaling at a concentration of 1:1000. The ALK protein was detectable by Western blots at relatively low temperatures (i.e. ≤51 °C). When the temperature increased, the ALK protein started to aggregate and gradually disappeared in the supernatant after centrifugation. In the event of substantial crizotinib—ALK binding, ALK was relatively protected and it remained in the supernatant at a relatively high temperature (i.e. ≥52°). Protein expression levels on western blots were quantified by densitometry analyses using the ImageJ software.

### Reagents, siRNAs and plasmids transfection

Crizotinib (PF-2341066), was purchased from Sigma-Aldrich (Oakville, Ontario, Canada). siRNAs targeting β-catenin (smart pool) as well as a scrambled siRNA control (Dharmacon) were transfected with Lipofectamine RNAiMAX Reagent (Life Technologies) at a final concentration of 40 nM. Human β-catenin-wt, ΔN47 β-catenin and ΔN89 *β-catenin*, all of which are in the pcDNA3 backbone, were purchased from Addgene (plasmids #16828, #19287 and #19288, respectively). The plasmid carrying *NPM-ALK* was a kind gift from Dr. S Morris, St. Jude Children’s Research Hospital (Memphis, TN, USA) and the *NPM-ALK* construct was cloned into the pcDNA3 vector (Invitrogen, Burlington, Ontario, Canada) as described previously^66^.

### Western blot assay

Cell lysates were prepared with RIPA buffer (Cell Signaling) supplemented with protease inhibitors (Calbiochem, San Diego, CA, USA) and phosphatase inhibitors (Calbiochem). Proteins were separated on 10% SDS-PAGE and transferred to PVDF membrane (Millipore). The membranes were blocked in 5% non-fat milk and incubated with antibodies against ALK and β-catenin (both antibodies were purchased from Cell Signaling Technology), and vinculin (Santa Cruz). Next, the blots were washed and incubated with Anti-Rabbit IgG, HRP-linked Antibody (Cell Signaling) or Anti-mouse IgG, HRP-linked Antibody (Cell Signaling) and detected with ECL Western Blotting Substrate (Pierce).

### Statistical analysis

All the statistical analyses were performed using the GraphPad Prism 5.1 software. Student *t* test was used to compare two independent groups. *P* values were considered statistically significant at less than 0.05. Results are presented as mean ± standard deviation.

## Electronic supplementary material

Supplementary data
