# Supplementary material for: High expression of β-catenin contributes to the crizotinib resistant phenotype in the stem-like cell population in neuroblastoma
Source: Sci Rep. 2017 Dec 4;7:16863. doi: 10.1038/s41598-017-17319-9 (PMC5715105; doi:10.1038/s41598-017-17319-9)
Supplement: Supplementary file 1 — Supplementary data [file 41598_2017_17319_MOESM1_ESM.pdf]

Supplementary Data

High expression of β-catenin contributes to the crizotinib resistant phenotype in the stem-like cell population in neuroblastoma

Abdulraheem Alshareef[1,2], Nidhi Gupta[1], Hai-Feng Zhang[1], Chengsheng Wu[1], Moinul Haque[1], Raymond Lai[1,3,4]*

[1]Department of Laboratory Medicine and Pathology, University of Alberta, Edmonton, Alberta, Canada;

[2]Department of Applied Medical Sciences, Taibah University, Almedinah, P.O. Box 41477, Saudi Arabia;

[3]Department of Oncology, University of Alberta, Edmonton, Alberta, Canada;

[4]*DynaLIFE* Medical Laboratories, Edmonton, Alberta, Canada

***To whom correspondence should be addressed:**

Raymond Lai, MD, PhD, Department of Laboratory Medicine and Pathology, Cross Cancer Institute and University of Alberta, 11560 University Avenue, Room 2338, Edmonton, Alberta T6G 1Z2, Canada; E-mail: rlai@ualberta.ca.

Supplementary Figure 1

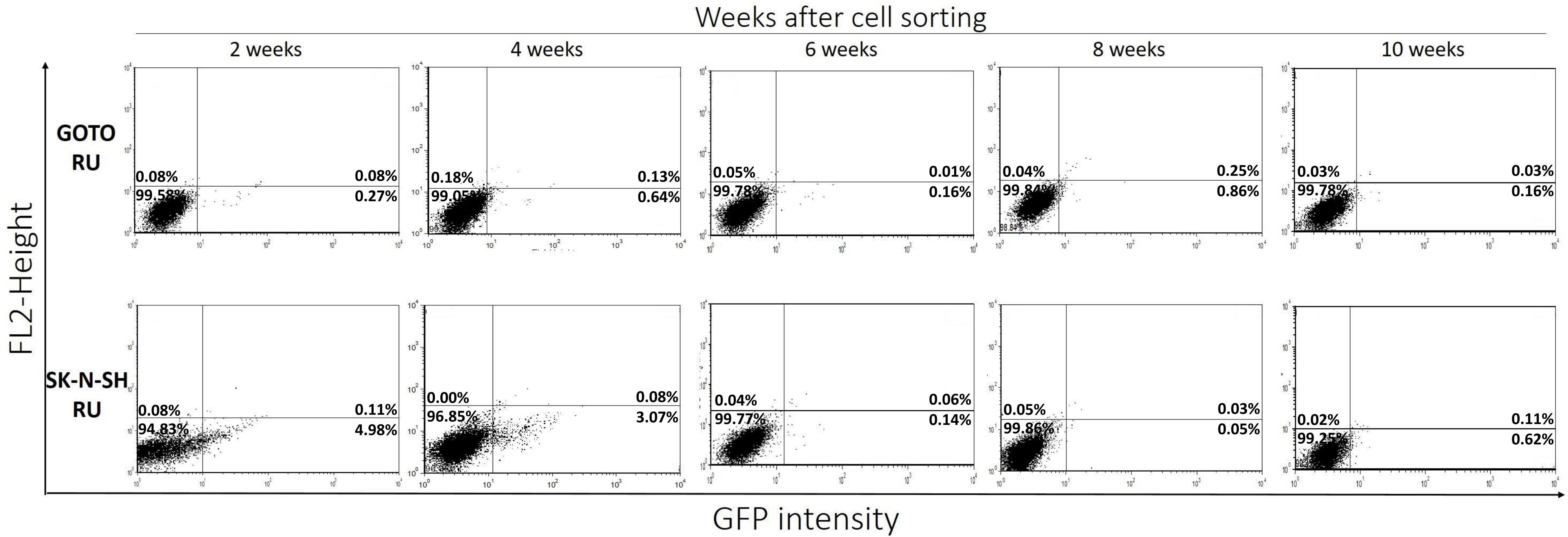

Supplementary Figure 2

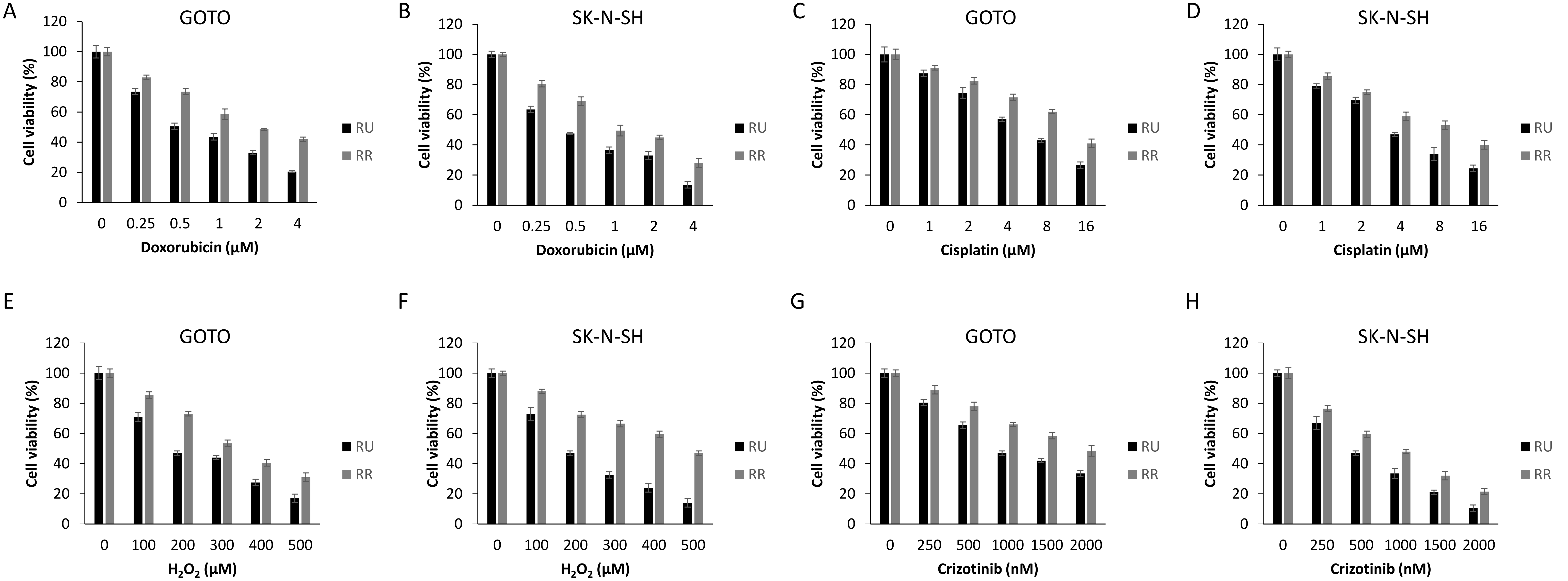

Supplementary Figure 3

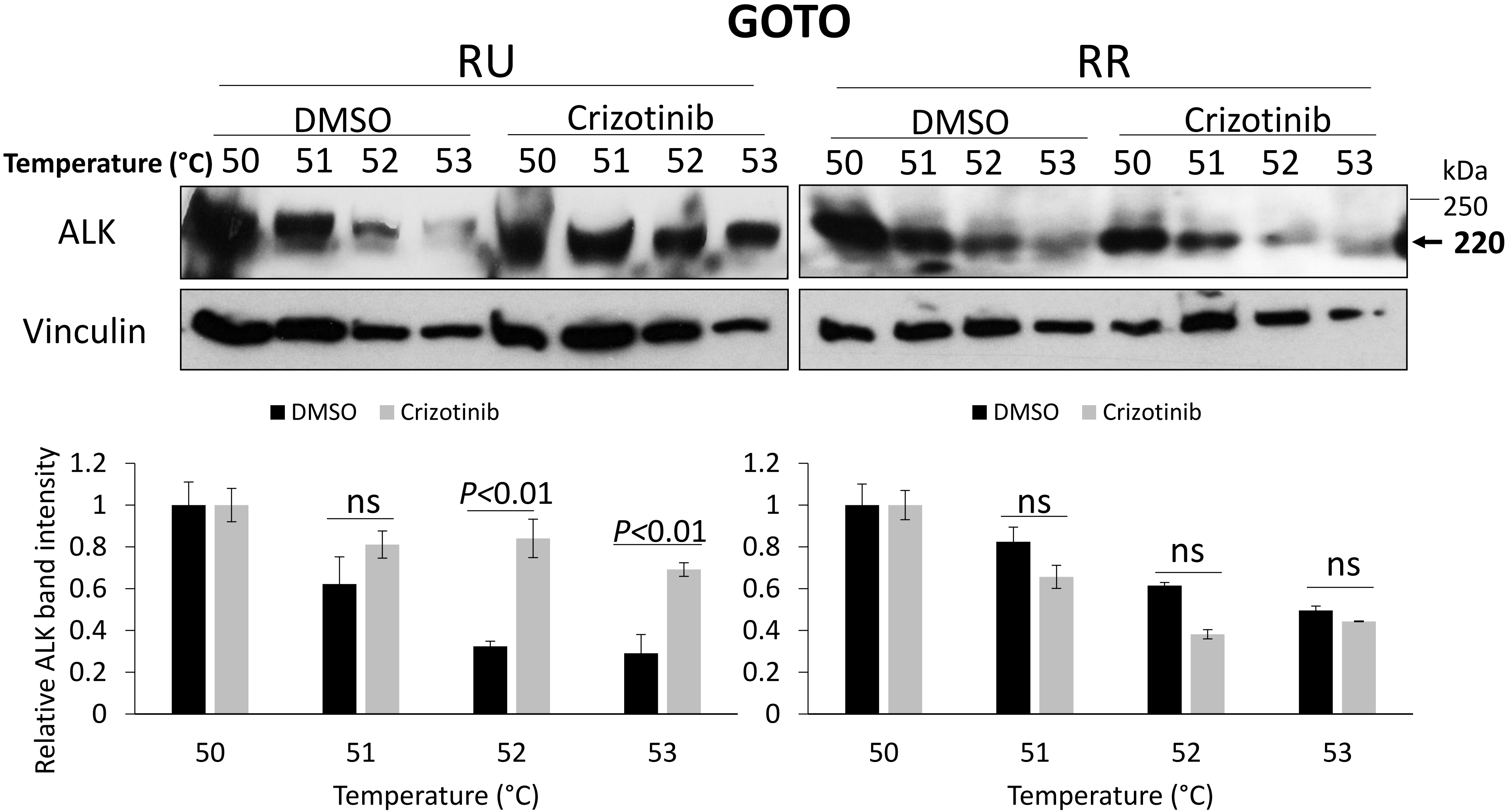

Supplementary Figure 4

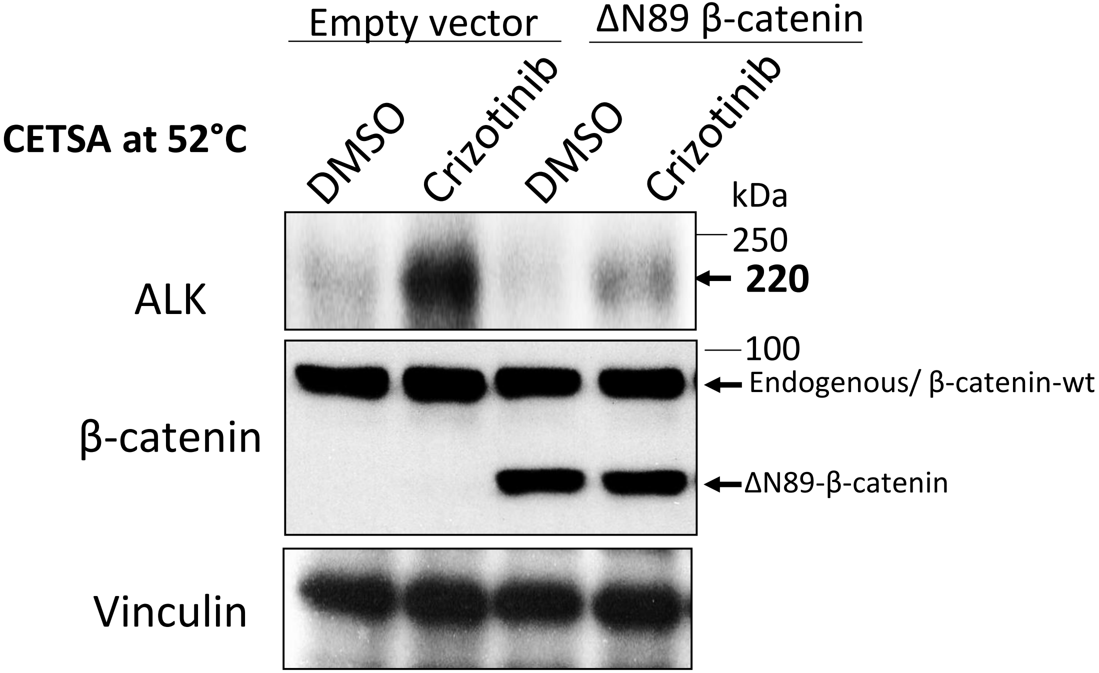

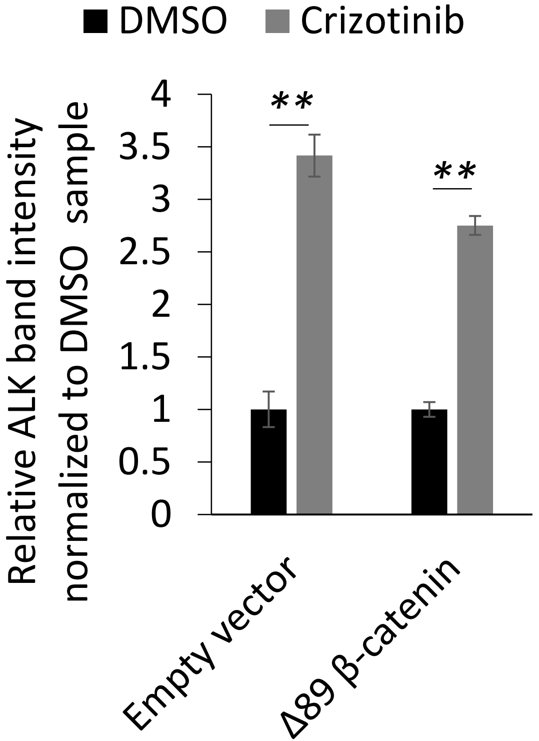

Supplementary Figure 5

GP293 cells transfected with full-length ALK^{wt}

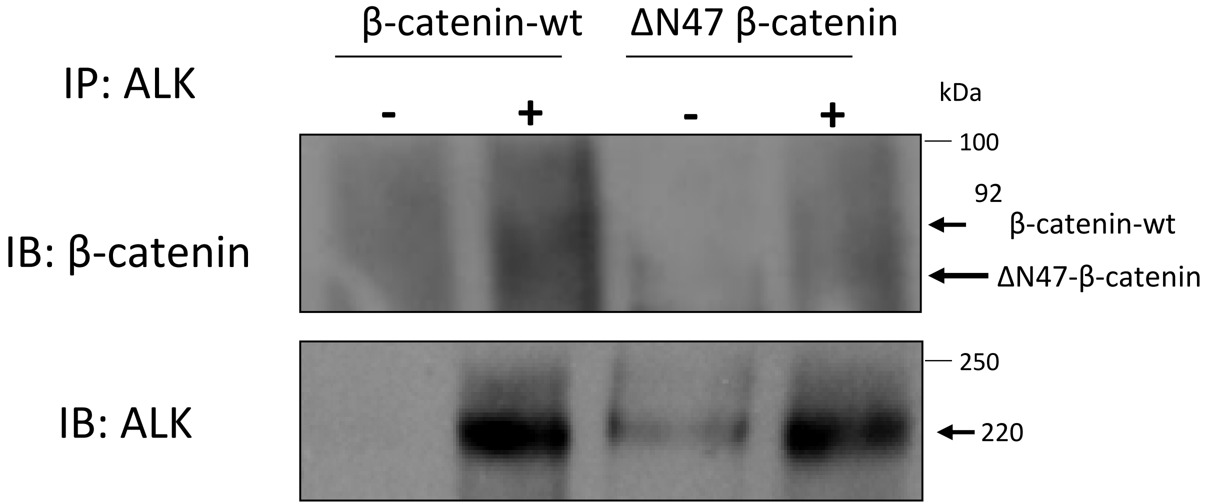

Supplementary Figure Legends

Supplementary Figure 1. RU cells do not convert into RR cells. Flow cytometry analysis was performed to assess GFP expression in purified RU cells derived from GOTO and SK-N-SH. No appreciable emergency of GFP-positive cells was found in both cell lines over 10 weeks.

Supplementary Figure 2. RR cells are significantly resistant to chemotherapeutic agents, oxidative stress and crizotinib. Purified RU and RR cells derived from 2 NB cell lines were subjected to two chemotherapeutic agents (doxorubicin and cisplatin) (**A-D**), oxidative stress (i.e. H_2O_2) (**E & F**) and crizotinib (**G & H**). The number of viable cells were counted at 72 hours after the treatment. Triplicate experiments were performed and a representative experiment is illustrated.

Supplementary Figure 3. RR cells derived from GOTO demonstrate no crizotinib—ALK binding. CETSA was performed to compare crizotinib—ALK binding ability between RU and RR cells. RU and RR cells derived from GOTO were treated with DMSO or 500 nM crizotinib for 6 hours. Representative ALK western blots are shown on the upper panel. Vinculin level was blotted as a loading control. The densitometry quantification data from 3 independent experiments are shown on the lower panel. All data are presented as mean ± SD. Student's *t* test was performed.

Supplementary Figure 4. Enforced ΔN89-β-catenin maintained crizotinib—ALK binding in SK-N-SH-RU cells. CETSA was performed to compare crizotinib—ALK binding ability in RU that were transfected with either empty vector or ΔN89-*β-catenin*. Cells were treated with DMSO or 500 nM crizotinib for 6 hours. Representative ALK western blots are shown on the left panel.

CETSA assay was performed at 52°C. Vinculin level was blotted as a loading control. All data are presented as mean ± SD, $*P<0.05$, $**P<0.01$, Student's *t* test.
